# 3D Finite Element Study on: Bar Splinted Implants Supporting Partial Denture in the Reconstructed Mandible

**DOI:** 10.3889/oamjms.2016.027

**Published:** 2016-02-09

**Authors:** Mohamed El-Anwar, Rami Ghali, Mona Aboelnagga

**Affiliations:** 1 *National Research Centre, Mechanical Engineering, Giza, Egypt*; 2 *Faculty of Dentistry, Ain Shams University, Removable Prosthodontics, Cairo, Egypt*

**Keywords:** Finite element method, Stress Distribution, Bar splinted implants, Reconstructed Mandible, Partial Denture

## Abstract

**AIM:**

This study aimed to estimate the stress patterns induced by the masticatory loads on a removable prosthesis supported and retained by bar splinted implants placed in the reconstructed mandible with two different clip materials and without clip, in the fibula-jaw bone and prosthesis using finite element analysis.

**METHODS:**

Two 3D finite element models were constructed, that models components were modeled on commercial CAD/CAM software then assembled into finite element package. Vertical loads were applied simulating the masticatory forces unilaterally in the resected site and bilaterally in the central fossa of the lower first molar as 100N (tension and compression). Analysis was based on the assumption full osseointegration between different types of bones, and between implants and fibula while fixing the top surface of the TMJ in place.

**RESULTS:**

The metallic bar connecting the three implants is insensitive to the clips material. Its supporting implants showed typical behavior with maximum stress values at the neck region. Fibula and jaw bone showed stresses within physiologic, while clips material effect seems to be very small due to its relatively small size.

**CONCLUSION:**

Switching loading force direction from tensile to compression did-not change the stresses and deformations distribution, but reversed their sign from positive to negative.

## Introduction

Mandibular defect is the defect affecting the mandibular integrity following surgical removal of oral neoplasm or trauma. It is debilitating and causes a significant impact on the patient’s quality of life. This is because segmental resection of the mandible leads to significant patient morbidity [1, 2]. It is in the form of loss of mandibular support to the teeth, tongue and lip. In turn, this leads to dysfunctional mastication, swallowing, speech, impaired airway protection and oral incompetence [3, 4].

Patients also suffer from disfigurement following segmental mandibulectomy because the mandible is an aesthetic landmark. The degree to which dysfunction and disfigurement occur depends on both the location of mandibular segment removed and the amount of soft tissue excised. Therefore, the overall goal of mandibular reconstruction is to restore the patient’s aesthetic deformity and functional loss that occur with this defect [5, 6].

Mandibular reconstruction and oral rehabilitation pose many challenges to the surgeon to restore function and esthetics. Many orofacial reconstruction strategies have been developed to provide an ideal bony reconstruction for later dental rehabilitation aiming to restore function and appearance to resemble the normal condition as closely as possible [5]. Wide variety of techniques and materials has been used to repair defects resulting from mandibular resection. In 1990, the use of bone grafting for mandibular defect repair was first described; where, all of these techniques had some degree of success but none was reliable enough to be routinely used [7].

Advances in microvascular surgical reconstruction techniques and the dental use of vascularized free bone grafts have broadened the possibilities for oral restoration for these patients, offering the ability to restore the hard and soft tissue orofacial defects and providing the suitable foundation for osseointegrated implant placement [8].

The fibula graft is reported to be preferred over other vascularized grafts as it is a stable one, due to its high cortical content which has as well high percentage of bone morphogenic proteins that osteoinductively promote the bone healing process [9, 10]. Fibula graft can be used to bridge gaps up to 25cm in length, as it is a long bone and has high mechanical resistance to pressure and torsion. Moreover, the fibula flap can be easily harvested with convenient sized blood vessels for anastomoses allowing rapid healing of vital flap. Implants inserted into the fibula grafts were studied and there had been no significant reduction of the success rate when compared to implants inserted into healthy mandibular bone [11].

On the other hand, the fibula free flap is limited in vertical height that is challenging for the final oral rehabilitation; either creating a significant step at the graft–mandible junction challenging placement of dental implants posing more stresses and unfavourable bending moment delivered to such implants or the fibula is aligned at the level of the alveolar crest thus not restoring the contour of the lower border of the mandible jeoparadizing aesthetic result [12]. The “double barrel” technique was described by Bahr in 1998 to overcome such problem in which a long fibular flap is halved and folded onto itself to increase the height of the “neomandible” [13].

Implant tooth supported partial overdenture are preferred over fixed options since the denture flange helps to improve the facial appearance, more posterior placement of the artificial teeth and better tongue management of food. Moreover, overdentures provide daily access for hygiene maintenance of the implant abutments to minimize periimplant soft tissue problems [14]. Dental implants used with implant supported partial overdentures improve retention, and stability where, masticatory performance is restored to presurgical levels, compared to conventional tissue borne ones particularly on the defect side [15, 16]. Compromise between retention, the need for stress distribution and maintenance of bone around the implants is a major factor affecting attachment selection [17].

Different attachment designs in maxillofacial prosthesis as splinted implants using resilient bar with clips, ERA, O-ring and OSO attachments are available. Although the bar with O-ring attachments resulted in more favourable stress distribution than either the bar-clip or the bar-ERA deign, the O-ring design was not as retentive as the other attachment systems used [17]. Bar-splinted dental implants supporting the overlay prosthesis allow better stress distribution and less prosthetic maintenance in comparison to non splinted implants. The incorporation of a clip with such prosthesis will improve its retentive quality. Clip attachments may be plastic or metallic preferably gold. Plastic clips take up more space, more prone to be dislodged, suffer more wear and tear and offer less retention in comparison to gold ones [18].

Recently, the finite element method (FEM) has been widely applied to prosthetic dentistry to predict stress and strain distribution at periimplant region, investigating the influences of implant and prosthesis designs, the magnitude and direction of load, bone mechanical properties as well as modeling different clinical scenarios [19-21].

As the stress distribution is an important factor for bone resorption during rehabilitation, the attachment system should present an adequate stress transfer to avoid bone resorption and improve treatment prognosis. Finite element analysis (FEA) was used to study the effect of different types of attachment systems on stress distribution; one study revealed that overdenture retained by unsplinted implants displayed stress concentration at both mesial and distal sides of the implants while for splinted implants stress concentration was observed at the distal side of the implant [22].

This study aimed to estimate the stress patterns induced by the splinted implants with bars prostheses and splinted implants with bars retained with plastic or metal (Gold) clips placed in the reconstructed mandible using FEA.

## Material and Methods

**Figure 1:**
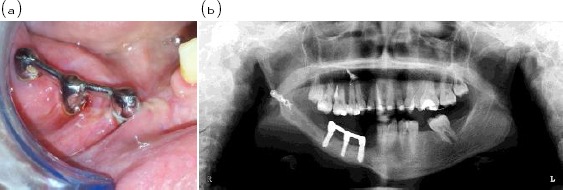
*(a) Showing intraoral view of three implants splinted with bar inserted in the right side of partially edentulous reconstructed mandible (b) radiographic view*.

A partially edentulous female patient having her mandible reconstructed with vascularized free fibula graft in the right segment of the mandible with the last standing tooth being the lower right canine was selected and a Cone Beam CT scanning of the patient was used to obtain an accurate geometric 3D model of the reconstructed mandible, Figure (1).

The construction of the printed reconstructed mandible model was divided into three steps; image acquisition of the 3D Cone Beam Computed Tomography scans-Imaging protocol of patient having mandible reconstructed with vascularized free fibula graft, construction of the 3D model using the Mimics software (Materialize Software Solutions, Leuven, Belgium. Version 10.01) through importing, threshold, mask creation and 3D reconstruction, and finally production of the printed model by the help of multi-jet modeling (thermal material application with UV curing) rapid prototyping machine (Invision Si_2_, 3D systems, Rock Hill, SC, USA, Present in the Central Metallurgical Research and Development Institute, Helwan, Egypt).

**Figure 2:**
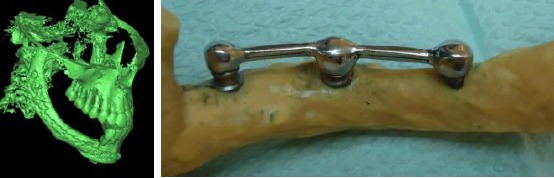
*The printed model of the reconstructed mandible (a) bone masking, (b) final prototype*.

Three conventional implants (Osseo-Link, Global Implant Solutions, LLC, MA, USA) were inserted into the fibula on the reconstructed mandible in the premolar molar area; anterior and middle implants were 5mm in diameter and 11 mm in length while the posterior implant was 3.5 mm in diameter and 11 mm in length. The length of the edentulous area was measured and 1.5 mm was left from the last tooth. The implants were placed in the resultant space equidistant from each other, thus the implant neck coincides with the crest of the ridge as in Figure 2. Three abutments were secured to the implants and then shortened that the future occlusal plane of the artificial teeth coincides with that of the natural ones. The plastic bar and wax pattern for dome shaped copings covering the abutments were cast and secured in place.

Rest seats for the double Aker clasp were prepared on the occlusal surface of the lower left first and second molars. Undercuts below the bar were blocked out using rubber base material. Mandibular impression was made in a stock tray using rubber base impression material for the fabrication of the partial overdenture which was then drawn. Clips were placed over the bar and relief of the fitting surface of the denture base was made for pick up of the clip.

In this study, two 3D FE models were constructed to simulate implant resection prosthesis placed in the reconstructed mandible supported and retained by bar splinted implants with and without clips. The printed model of the reconstructed mandible, three implants, bar, clips and mandibular partial overdenture were modeled in 3D as separate components (parts as; jaw bones, fibula, implant complex, bar, clips and overdenture).

Model components were modeled in 3D on commercial general purpose CAD/CAM software AutoDesk Inventor (Autodesk Inc., San Rafael, CA, USA, version 8.0). That each component was exported as SAT file before importing them into ANSYS (ANSYS Inc., Canonsburg, PA, USA).

Set of Boolean operations were carried out to assemble all the model components before meshing. The meshing software was ANSYS version 12 and the used element in meshing all three-dimensional models is 10-node tetrahedral structural solid element (SOLID187), which has three degrees of freedom (translations in the global directions) [23]. Mesh density compromise is an important parameter that, affects the results accuracy. The final mesh density of all modeled components is tabulated in Table 1.

**Table 1:** Number of Nodes and Elements

	Nodes	Elements
Fibula Cancellous Bone	23,742	110,487
Fibula Cortical Bone	4,611	14,339
3 x Implant-abutment Complex	41,108	212,828
Bar	5,123	20,475
2 x Clips	1,189	3,960
Overdenture	14,450	53,096
Mandible	14,219	65,187

**Figure 3:**
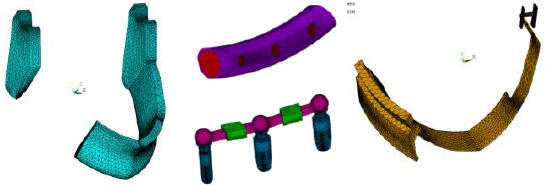
*Screen shots of ANSYS screens showing all parts of the model separately*.

**Figure 4:**
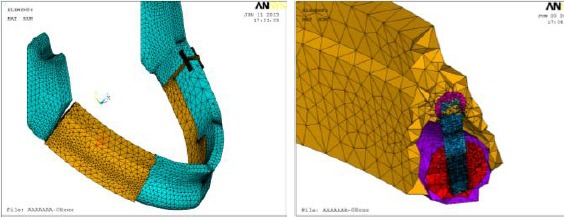
*Assembled model and sectional cut showing fibula, supporting system, and overdenture. 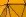
Overdenture; 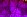
Coping; 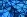
Implant; 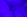
Fibula cortical; 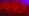
Fibula cancellous*.

Figures 3 and 4 show the meshed components of the model, where colors indicate different material properties. Where, all materials used in this model including alveolar bone, fibula, implants, bar, clips and overdenture were assumed to be isotropic, homogenous and linearly elastic. Modulus of elasticity and Poisson’s ratio of each model component were fed to FEA package (listed in Table 2).

**Table 2:** Material properties

Material	Young’s modulus [MPa]	Poisson’s ratio
Fibula Cancellous Bone	700	0.20
Fibula Compact Bone	14,000	0.40
3 x Implant/Abutment complex (titanium)	110,000	0.33
Bar (chrome-cobalt alloy)	218,000	0.33
2 x Clips (plastic)	3,000	0.28
2 x Clips (gold)	97,000	0.33
Overdenture (Acrylic resin)	3,000	0.35
Mandible (weighted average of alveolar cancellous and compact bones)	4,450	0.30

Frictional contact between prosthesis, clips, and bar was defined by the elements CONTACT 174 and TARGET 170 as surface to surface contact [23]. In addition, the model was fixed in place at TMJ top surface as a boundary condition, while loads were applied in a vertical direction (tissue ward and tissue away) perpendicular to the occlusal surfaces, at the central fossa of the lower first molar for each model as 100N [24]. The solid modeling and linear static analysis FEA were performed on a personal computer Intel Pentium Core 2 Duo, processor 3.0 GHz, 4.0GB RAM.

Four runs were performed on the model two for compressive and tensile load with using two plastic clips. While in the third run a different clips material (Gold clip) was evaluated under tensile loading, and the last one was performed without clips under tensile loading.

## Results

Finite element analysis resulted in a huge number of graphical illustrations of stresses, strains, and deformation distributions on each component in the studied model. Such results can be presented on the whole model and/or each component, showing color variation from dark blue (represent minimum value) to dark red (represent maximum value).

**Figure 5:**
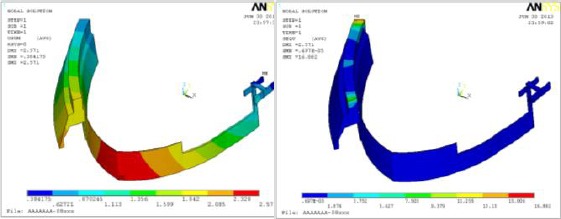
*Sample of overdenture results, showing (left) total deformation and (right) Von Mises stress distribution under compressive loading of 100N*.

As illustrated in Figure 5, total deformation and Von Mises stress distribution on the overdenture under compressive loading of 100N. Maximum value of Von Mises stress appeared under the applied load, while the maximum value of deformation was found at lingual side (downward or upward) according to the applied load direction (compression or tensile respectively).

**Figure 6:**
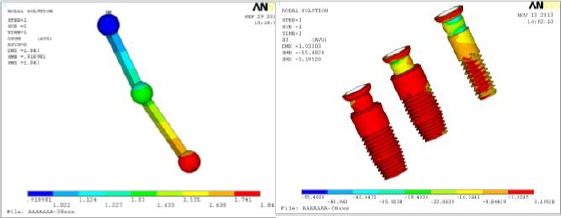
*Using gold clips under tensile loading showed (left) Bar total deformation distributions (right) compressive stress distribution on the three implants*.

Harder clips material (gold) receives higher level of stresses than the softer one (plastic). While, the metallic bar connecting the three implants is insensitive to the clips material. In this study, typical implant complex behavior was obtained, that it showed maximum stresses at implant neck at the connection with cortical bone as in Figure 6-b. The first and second mesial implants away from TMJ showed higher stresses and deformations than the third distal implant.

**Figure 7:**
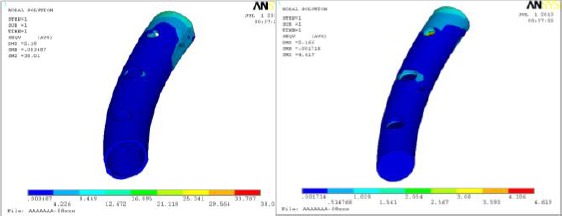
*Fibula (left) Compact (right) cancellous bone Von Mises stress distribution under tensile loading*.

Fibula compact and cancellous bones behavior are presented as Von Mises stress distributions in Figure 7. Similar distribution can be obtained in compressive loading, but generally both showed Von Mises stress values within physiologic limits.

**Figure 8:**
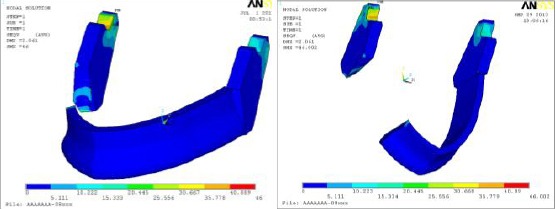
*Jaw bone behavior under tensile loading with two clips materials (left) plastic as soft material and (right) gold as hard material*.

Finally, the mandible is insensitive to clips material as in Figure 8 and showed Von Mises stress values within physiologic limits in all case studies included within this research. Finally summary of most important results obtained in this study are tabulated in Table 3.

**Table 3:** Prosthesis behavior summary

	*3 Implants*		*Bar*		*Clips*		*Over Denture*		Fibula Cancellous		Fibula Compact	
*Run*	U_sum_	S_von_	U_sum_	S_von_	U_sum_	S_von_	U_sum_	S_von_	U_sum_	S_von_	U_sum_	S_von_
*With plastic clip Compression*	1.93	44.24	1.84	76.13	1.65	2.55	2.57	16.88	2.16	4.61	2.18	38.01
*With plastic clip Tension*	1.93	44.24	1.84	76.13	1.65	2.55	2.57	16.88	2.16	4.61	2.18	38.01
*Without clip Tension*	1.93	44.24	1.84	76.13			2.57	16.88	2.16	4.61	2.18	38.01
*With gold clip Tension*	1.93	44.21	1.84	76.56	1.66	2.70	2.57	16.89	2.17	4.62	2.18	37.99

Where: U_sum_: total deformation in mm

S_von_: Von Mises stress in MPa

## Discussion

FEA was used for studying different designs for prosthetic rehabilitation of reconstructed mandibles. That, the stress level on the bone graft can be determined and quantified in every portion of the reconstructed mandible in addition to evaluation of prosthetic components deformations by the help of such studies [24-27].

In this study, the fibula free flap was utilized as bone graft due to long edentulous span that offers extensive periosteal blood supply and multiple osteotomies, to be made helping to precisely adapt the bone flap to the recipient site and to replicate the contour of the resected mandible [28]. Furthermore, the success rate of implants inserted into the fibula grafts shows no significant difference from those placed into healthy mandibular bone [29, 30].

Three implants were used for better stress distribution, where the anterior implant was placed 1.5mm from the lower right canine to provide support near that terminal tooth thus preventing excessive stresses to be delivered to it. Furthermore, this anterior implant acts as terminal rest for the lingual plate major connector instead of placing a terminal canine rest on the lower right canine.

The remaining space of the edentulous span after the anterior implant was divided for equidistant placement of the implants helping with more favourable anteroposterior distribution of force. Bar helps with wide anteroposterior distribution of forces, provides even support over a great surface area, thus helping to reduce the load on the soft tissues. Bar/Clip attachment helps with abutments splinting, offers high retention capacity and minimizes prosthesis movement during function. Two clips were placed one over each segment of the bar.

Overdenture overall volume and stiffness are negligibly affected by removing small volume to place the clips inside. Clips are usually made from plastic material which is too close to the overdenture resin material. That is why using clips or not is insignificant. Similarly, using harder clips material as gold has a negligible effect on overdenture behavior. Although there is a considerable difference in friction coefficient between, the plastic clips and gold clips, and the metallic bar the clips design do not depend on friction in fixing the overdenture on the metallic bar. Therefore gold clips are not recommended due to the high cost without obtaining effective difference.

On the other hand, plastic attachments housed in the prosthesis wear rapidly rendering such attachments becoming ineffective and leading to bone loss around the implant adjacent to the defect. Thus frequent replacement for such attachments is needed a factor that contributes to increased cost. Furthermore, Plastic clips take up more space, more prone to be dislodged, suffer more wear and tear and offer less retention in comparison to gold ones [31- 33].

Fibula cancellous and compact bones, in addition to jaw bone are safe under the expected loads bilaterally in the central fossa of the first molar as 100N (tension and compression).

Unilateral load of 100 N was applied in tissueward and tissueaway direction to evaluate the stress pattern induced in the reconstructed mandible in the two models. The load was applied at the central fossa of the lower first molar in vertical direction mimicking the effect of load in centric occlusion [27].

If the assumption of full osseointegration between different types of bones, and between implants and fibula existing, the levels of generated stresses on bones will be far enough from endurance limit. Thus such system for mandible reconstruction is optimal, and it behaves well under the expected loads [18-21].

Within the limitations of the present study, there was a stress concentration in the region of the grafted bone/mandible interface and at the region of the sigmoid notch, coronoid process and condylar neck on the reconstructed side as proven by a previous study [19]. This can be explained in the view of size discrepancy between grafted bone and native mandible. Thus, to obtain better fixation and healing after bone grafting and to reduce the influence of stresses, osteosynthesis plates or supplementary fixation in the inferior border of reconstructed mandible should be applied [19, 34]. Due to discontinuity of the stress line in the mandible reconstructed with the fibula, the stresses on the healthy side were less than on the defective side [19].

The principal stresses obtained were the same on comparing the absence and presence of the plastic clip attachment. This may be attributed to the similarity of clips/overdenture material in addition to the splinted implants that were assumed to be 100% osseointegrated with the surrounding bone; that these implants can withstand all the stresses within its physiologic limit regardless the use of the clip/bar attachment.

When the results were analyzed regarding the stress distribution in all loading situations, the highest stresses were concentrated in the bar followed by the cortical bone around the implant neck as proven in other studies [19, 20]. Similar conclusions of previous studies regarding the location of the maximum stresses on the cortical layer is closely related to the material properties assigned to bone; as the cancellous core has modulus of elasticity less than that of the cortical layer, the implants were only supported by cortical bone which would absorb most of the stresses, while the reaction forces of the cancellous bone upon the loaded implant would be underestimated [35, 36].

In the view of the mechanical principle stating that when two materials of different moduli are placed together with no intervening material and one is loaded, a stress contour will be observed where the two materials come into contact. These stress contours are of the greatest magnitude near the point of the first contact [37].

Regarding this principle, the importance of using the bar to splint the implants is observed, that the bar will bear the greatest stresses as demonstrated in the results. This prevents the greatest stresses to be developed around the implant neck and subsequent bone resorption; thus implant success rate is improved especially with the irregular bending patterns that arise during mastication due to asymmetric nature of reconstructed mandibles [37].

The forces resulting from occlusal loading is damaging to the implants, thus it is preferable to splint the implants with a rigid bar to direct most of the occlusal forces along the long axis of the implants. Additionally, as the modulus of elasticity of the plastic clip is similar to that of acrylic resin, the values of the stresses delivered to the reconstructed mandible did not change in the analysis of the two models with/without plastic clips.

Despite the great stresses developed in the reconstructed part and at its interface with the native mandible, it is worth to be mentioned that constant recurrence of increased stresses during the ossification process results in thickening of the reconstructed parts according to Wolff’s law; which says that bone remodels itself to shapes that are suited to bear external stresses [38-41].

Within the limitations of this study, the following conclusions can be drawn:

- Using clips or not has a negligible effect on all other parts of the studied model. In addition clips material rigidity did not influence the jaw bones, implant complex, and metallic bar.
- Switching loading force from tensile to compressive did not change stresses and deformations distribution, but it reverses their sign from positive to negative and vice versa.
- Fibula and jaw bones showed safe behavior with the assumption of full osseointegration between different types of bones, and between implants and fibula.

## Ethical approval

This research was approved by Research Ethics Committee (REC) of Medical Faculty, Ain Shams University (FMASU R 33/2015, September 13, 2015).
